# A full-term pregnant woman with secondary Evans syndrome caused by severe coronavirus disease 2019: a case report

**DOI:** 10.1186/s13256-021-03205-6

**Published:** 2021-12-13

**Authors:** Damai Santosa, Muchlis A. U. Sofro, Farida, Nurvita Nindita, Eko A. Pangarsa, Budi Setiawan, Daniel Rizky, Catharina Suharti

**Affiliations:** 1 Department of Hematology Medical-Oncology, Dr. Kariadi General Hospital, Semarang, Indonesia; 2 Department of Tropical-Infection, Dr. Kariadi General Hospital, Semarang, Indonesia; 3 Department of Pulmonology, Dr. Kariadi General Hospital, Semarang, Indonesia; 4 Department of Obstetrics and Gynecology, Dr. Kariadi General Hospital, Semarang, Indonesia; 5 Faculty of Medicine, Diponegoro University, Semarang, Indonesia

**Keywords:** Evans syndrome, COVID-19, C-section delivery

## Abstract

**Background:**

In this report, we describe a very challenging case of a patient with secondary Evans syndrome caused by severe coronavirus disease 2019 infection in a pregnant full-term woman.

**Case presentation:**

A 29-year-old full-term pregnant Indonesian woman presented with gross hematuria, dry cough, fever, dyspnea, nausea, anosmia, and fatigue 5 days after confirmation of coronavirus disease 2019 infection. Laboratory examinations showed very severe thrombocytopenia, increased indirect bilirubin, and a positive direct Coombs’ test. From peripheral blood, there was an increased number of spherocytes, which indicated an autoimmune hemolytic process. Antinuclear antibody and anti-double-stranded DNA test results were negative, and her virology serological markers are also negative for human immunodeficiency virus, cytomegalovirus, and hepatitis B and C. Despite aggressive treatment with platelet transfusion, high-dose steroid, and thrombopoietin receptor agonists, the platelet count did not recover, and a speculative cesarean delivery had to be done with a very low platelet count.

## Background

Coronavirus disease 2019 (COVID-19) has been a devastating pandemic all across the world since late 2019. Evans syndrome (ES) is an uncommon disorder characterized by the destruction of red blood cells and platelets by the patient’s antibodies [1]. It has been reported as one of the COVID-19 complications, and immune dysfunction also usually occurs in pregnancy [2, 3]. We report a challenging case of a full-term pregnant Indonesian woman with severe COVID-19 complicated by secondary ES with a successful outcome. Limitations of the availability of the medications in our country might halt the treatment progression; hence, we had to decide on a threatening C-section delivery procedure with a platelet count of 1 × 10^9^/L. To date, there have not been any published data on COVID-19 complicated with ES and full-term pregnancy.

## Case report

A 29-year-old Indonesian woman was admitted to Dr. Kariadi General Hospital with confirmed COVID-19 for 5 days. She was gravida 2 para 1 abortus 0 (G2P1A0), pregnant 39 weeks, and presented with gross hematuria, dry cough, fever, dyspnea, nausea, anosmia, and fatigue. She is a housewife and had no history of previous illnesses or trouble during her first pregnancy. She also routinely visited antenatal care monthly. There was no history of hematological abnormalities in her family. There were no imminent delivery signs. On admission, she was fully conscious, her vital signs were blood pressure of 109/77 mmHg, heart rate 89 beats per minute, respiratory rate of 28 breaths per minute, body temperature of 37.8 °C, and 100% blood saturation level with nasal cannula of O_2_ 2 L per minute. Her baby’s estimated weight is 4.000 g with a heart rate of 142 beats per minute, and there were no signs of fetal distress.

**Fig. 1:**
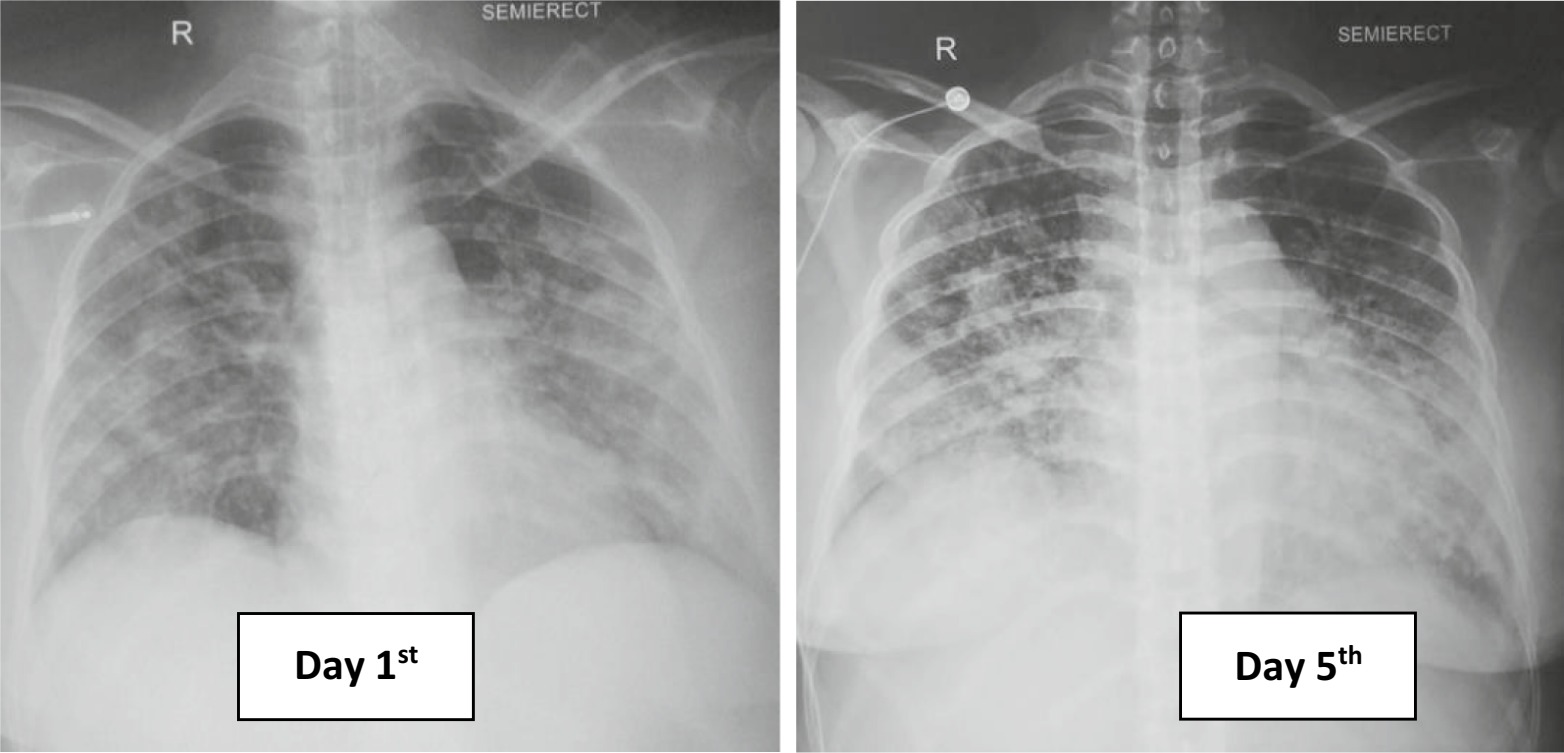
Comparison of chest X-ray within the treatment

**Fig. 2:**
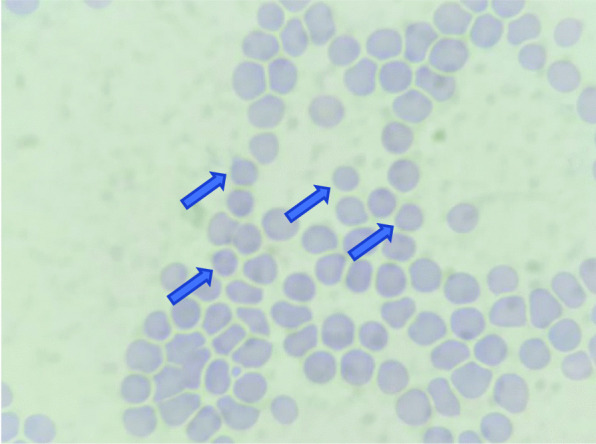
Spherocytes (blue arrows)

Her chest X-ray showed bronchopneumonia on both sides of the lung (Fig. 1). Her blood tests on admission revealed: hemoglobin (Hb) 10 g/dL; white blood cell count (WBC) 5.8 × 10^9^/L with neutrophil–lymphocyte ratio (NLR) of 11; lymphocyte 696 cells/µL, platelet count 2 × 10^9^/L; *P*/*F* (PaO_2_/FiO_2_) ratio 200; C-reactive protein (CRP) 7.77 mg/L; procalcitonin 0.35 ng/mL; prolonged activated partial thromboplastin time (aPTT) 54.9 (36.8) seconds; d-dimer level, 1.400 ng/mL; fibrinogen level 333 mg/dL; urinalysis showed proteinuria 2+, gross hematuria, and pyuria; renal, electrolyte, and liver functions were within normal limits; peripheral blood smear revealed that there were spherocytes (Fig. 2), which may indicate an autoimmune hemolytic process, and her virology serological markers were negative for human immunodeficiency virus (HIV), cytomegalovirus, and hepatitis B and C.

The patient was initially treated for COVID-19 with remdesivir 100 mg daily, moxifloxacin 400 mg daily, dexamethasone 6 mg daily, tranexamic acid 500 mg three times per day, *N*-acetylcysteine, vitamin C, D3, and zinc, and she was given 8 units platelet transfusion and 1 unit concentrate. After the transfusion, the platelet count decreased even further to 3 × 10^9^/L and was followed by moderate anemia Hb 9.1 g/dL caused by gross hematuria. On the third day, her condition deteriorated with dyspnea, and hematuria worsened; she was oxygenated with a high-flow nasal cannula (HFNC) 60 L per minute, and fetal distress signs were found. A very high-risk emergency delivery with cesarean section was performed, and platelet, fresh frozen plasma transfusions, and tranexamic acid were administered to control the bleeding. Periprocedurally, there was approximately 1 L of bleeding, and afterward, the patient was transferred to the intensive care unit (ICU). Her baby’s weight was 4.280 g, with an APGAR score of 8.

In ICU, she had overt disseminated intravascular coagulation with hematemesis, melena, and hematuria; her Hb level dropped to 5.8 g/dL, and the d-dimer level increased to more than 5000 mg/dL. During ICU hospitalization, she developed severe acute respiratory distress syndrome with a *P*/*F* ratio of 97, and she was on mechanical ventilation support. Antinuclear antibody (ANA) and anti-double-stranded DNA tests were negative, blood and urine culture was sterile, and direct Coombs’ test was positive. Bilirubin total/indirect/direct bilirubin was elevated at 4.6, 2.9, and 1.7 mg/dL, respectively. Reticulocyte count was 1.9%, d-dimer level was elevated to 5580 mg/dL, and serial chest X-ray showed pulmonary edema and worsened bronchopneumonia. Convalescent plasma, dexamethasone injection 5 mg twice per day, eltrombopag 50 mg daily, and diuretic were administered on day 5. On the seventh day, her condition was improved, the bleeding stopped, and the *P*/*F* ratio improved to 160. However, there were still no signs of platelet and hemoglobin level recovery, dexamethasone dosage was increased to 40 mg daily, and hydroxychloroquine 200 mg was administered daily due to the possibility of the diagnosis of systemic lupus erythematosus (SLE) with negative ANA test.

**Figure 3.**
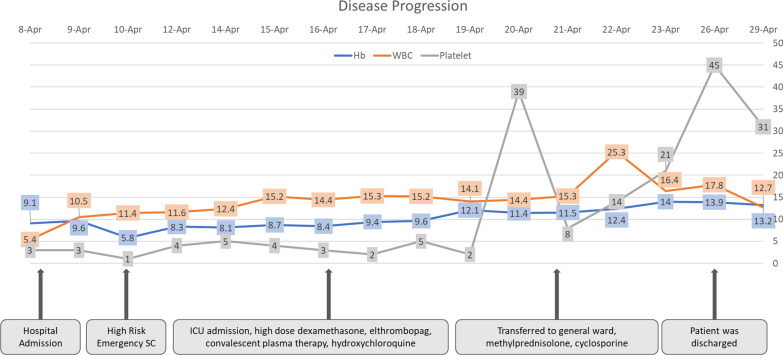
Patient’s treatment timeline

On the 11th day, her condition was improved and the bleeding has stopped with a platelet count of 5 × 10^9^/L with a no longer prolonged aPTT, and chest X-ray showed improvement. The HFNC flow was then tapered off, and she was transferred to the general ward. Because there was no improvement in platelet count with current treatment, we substituted dexamethasone with methylprednisolone 125 mg twice daily and added cyclosporin 50 mg twice daily. From the abdominal ultrasonography, there were no abnormalities. Surprisingly, on the 13th day, platelet count was dramatically increased to 39 × 10^9^/L. Afterward, for the remaining days of her hospitalization, the patient was stable, and she was discharged on the 19th day with a platelet count of 45 × 10^9^/L (Fig. 3). She received transfusion of a total of 2 units of platelet concentrate, 16 units of platelet, 5 units of leukodepleted packed red cells, 4 units of fresh frozen plasma, and 2 units of convalescent plasma. On follow-up, her ANA profile result was negative. There were no adverse events reported due to all of the medications. After 2 months of COVID-19 seroconversion, she was in a healthy condition, her hemoglobin and platelet levels returned to normal range, and all the medications were ceased. In conclusion, she was diagnosed with secondary Evans syndrome.

## Discussion

Here we describe a successful case of a full-term pregnant woman with secondary ES caused by severe COVID-19 infection. Although a similar case of COVID-19 complicated with ES was reported before [2], to our knowledge, this is the first documented example of a pregnant COVID-19 woman with platelet count 1 × 10^9^/L who underwent a successful cesarean section delivery with both mother and baby safe. Cesarean delivery had to be done because of the increased risk of fetal distress and the patient’s worsened condition although the considered safe platelet count for this procedure is above 50 × 10^9^/L [4].

Low platelet count is a common feature of COVID-19 infection and has been considered a poor prognostic factor [5–7]. Although most cases are associated with mild thrombocytopenia, a severely low platelet count was also associated with more severe disease and also increases the mortality rate [7]. Evans syndrome (ES) is a rare and chronic autoimmune disease characterized by autoimmune hemolytic anemia and immune thrombocytopenic purpura with a positive direct anti-human globulin test. ES is classified as primary (idiopathic) and secondary, with the frequency in patients with autoimmune hemolytic anemia being higher than 50%. [1]

Several authors have introduced various pathophysiological pathways of ES: first, deficiency of cytotoxic T-lymphocyte-associated antigen 4 (CTLA-4) and lipopolysaccharide-responsive beige-like anchor (LRBA) protein that plays a very important role in immune homeostasis; second, decreased CD4/CD8 ratio, decreased level of T helper with increased T cytotoxic activation resulting in stimulation of B-cell reactivity against blood cells; and third, a deficit of tripeptidyl peptidase 2 (TPP2) associated with the presence of antinucleolar, anticytoplasmic, and antinuclear antibodies. All of these mechanisms alter the body immunologic response observed in this syndrome [1].

Although the precise pathogenesis of how COVID-19 influences immune dysregulation is still unclear, recent studies have shown this process to be precipitated by COVID-19 infection [8]. Studies have found that regulatory T cells (Tregs), which play a specific role in repressing the immune response, are decreased even more in severe COVID-19 cases [9–11]. With this immune dysregulation, the cytotoxic CD8^+^ T cells also become activated and directly cause hematologic abnormalities [10, 12]. In addition, it has been determined that infections may worsen the pathogenesis of its therapeutic response to blood transfusions; in this case, the patient received a total of 18 units of platelet transfusion [13]. Platelet and fresh frozen plasma (FFP) transfusions were administered for this patient as a result of active bleeding and severely low platelet count and prolonged aPTT [14]. However platelet transfusions have not resulted in improvement of platelet count in this case, which also might be due to antiplatelet antibodies on the surface of transfused platelets [15].

Other various mechanisms of hematologic abnormalities in COVID-19 are: (1) direct infection of the virus to bone marrow and inhibition of the synthesis of blood cells, with cytokine storms also having the potential to disrupt the bone marrow progenitor cells and decrease the platelet count even further; (2) immunothrombosis, which has been one of the most common incidents in coagulopathy abnormalities in COVID-19 that also plays a role in platelet destruction; and (3) aggregation of platelets in vasculature, which may end with microthrombi and platelet consumption [16, 17].

However low platelet count in pregnancy is not an uncommon event and is usually caused by gestational thrombocytopenia, HELLP syndrome (hemolysis, elevated liver enzymes, low platelet count), pre-eclampsia/eclampsia, or acute fatty liver of pregnancy [3]. Autoimmune disorder in pregnancy might also be influenced by the translocation of cells between fetus and mother. This could be followed by the presence of fetal microchimerism, which is acquired by the mother during pregnancy. CD4^+^ T-cell cytokine profile also has a very specific role in autoimmunity in pregnancy. T-helper (Th)2 and Th17/Th2 cells are abundant in the circulation of pregnant women. These cells are capable of inciting an autoimmune response by influencing the T-cell cytokine-mediated responses during the gestation period [18].

From this case, differential diagnoses such as HELLP syndrome, atypical hemolytic uremic syndrome (aHUS), and thrombotic thrombocytopenic purpura (TTP) could be excluded, because there were no schistocytes from her blood smear examination, indicating that there was no thrombotic microangiopathy [19]. A positive test of Coombs’ test also indicated a hemolytic process, which was not found from the other differential diagnosis.

From a systematic review, a total of 45 COVID-19 patients with immune thrombocytopenia (ITP) were analyzed, with 71% of them having moderate to severe COVID-19 illness and most of them having a normal platelet count in a post-recovery period and diagnosis of a secondary ITP [12]. In this case, the patient had a full hematological recovery after 2 months of the COVID-19 infection, which supported the diagnosis of secondary Evans syndrome.

Most immune-associated thrombocytopenia cases were treated with intravenous immunoglobulin (IVIG) only, followed by glucocorticoids alone in and thrombopoietin receptor agonists (TP-RA) with or without combinations [12]. In our case, we gave dexamethasone per the protocol and noted no improvement. Although this is an indication for IVIG in a few cases [20, 21], there were limitations due to its cost and it not being widely available in our center. TP-RA and cyclosporine are drugs commonly used in later management of ITP [22]. We used these three combinations of glucocorticoid, TP-RA, and cyclosporine for this patient, resulting in platelet count improvement on day 13. A similar notable improvement in platelet count with TP-RA administration for 44 days was also shown in a case of ITP with COVID-19 [17].

Much evidence has shown that thrombosis is a common COVID-19 complication, and it has been associated with a high d-dimer level. Thromboprophylaxis was highly recommended in all hospitalized COVID-19 patients, especially for those who suffer from acute respiratory distress syndrome [23, 24]. In this patient, the condition is also worsened by disseminated intravascular coagulation (DIC), which may further decrease the platelet count. Although thromboprophylaxis was recommended for hospitalized COVID-19 patients, active bleeding in this patient was the absolute contraindication for this approach.

## Conclusion

Along with the improvement of clinical symptoms of COVID-19, our patient’s hematologic parameters also recovered, which was concluded as an autoimmune process of COVID-19. To our knowledge, we present the first case of Evans syndrome in a full-term pregnant woman with COVID-19. A combination of glucocorticoid, TP-RA, cyclosporine, and convalescent plasma seems to be effective for this patient. The crucial decisions regarding C-section delivery and choice of medications had to be made on a case-by-case basis by an experienced multidisciplinary team.

## Data Availability

Not applicable.

## Abbreviations

ES: Evans syndrome
CTLA-4: Cytotoxic T-lymphocyte-associated antigen 4
LRBA: Lipopolysaccharide-responsive beige-like anchor protein
C-section: Cesarean delivery
NLR: Neutrophil–lymphocyte ratio
CRP: C-reactive protein
aPTT: Activated partial thromboplastin time
ICU: Intensive care unit
TTP: Thrombotic thrombocytopenia
HUS: Hemolytic uremic syndrome
Tregs: Regulatory T cells
ITP: Immune thrombocytopenia
IVIG: Intravenous immunoglobulin
TP-RA: Thrombopoietin receptor agonists
FFP: Fresh frozen plasma

## References

[1] Jaime-Pérez J, Aguilar-Calderón P, Salazar-Cavazos L, Gómez-Almaguer D. Evans syndrome: clinical perspectives, biological insights and treatment modalities. J Blood Med. 2018;9:171–84. Available from: https://www.dovepress.com/evans-syndrome-clinical-perspectives-biological-insights-and-treatment-peer-reviewed-article-JBM. 10.2147/JBM.S176144 PMC6190623 30349415

[2] Li M, Nguyen CB, Yeung Z, Sanchez K, Rosen D, Bushan S. Evans syndrome in a patient with COVID-19. Br J Haematol. 2020;190(2):e59-61. 10.1111/bjh.16846 PMC7276874 32420629

[3] McCrae KR. Thrombocytopenia in pregnancy. Thrombocytopenia. 2006;11(1):265–74.

[4] Estcourt LJ, Malouf R, Doree C, Trivella M, Hopewell S, Birchall J. Prophylactic platelet transfusions prior to surgery for people with a low platelet count. Cochrane Database Syst Rev. 2017. 10.1002/14651858.CD012779. PMC5687560 29151812

[5] Ko J-H, Park GE, Lee JY, Lee JY, Cho SY, Ha YE, *et al*. Predictive factors for pneumonia development and progression to respiratory failure in MERS-CoV infected patients. J Infect. 2016;73(5):468–75. Available from: https://linkinghub.elsevier.com/retrieve/pii/S0163445316302092. 10.1016/j.jinf.2016.08.005 PMC7112644 27519621

[6] Wong RSM. Haematological manifestations in patients with severe acute respiratory syndrome: retrospective analysis. BMJ. 2003;326(7403):1358–62. 10.1136/bmj.326.7403.1358. PMC162124 12816821

[7] Zhu Y, Zhang J, Li Y, Liu F, Zhou Q, Peng Z. Association between thrombocytopenia and 180-day prognosis of COVID-19 patients in intensive care units: a two-center observational study. PLoS ONE. 2021;16:1–15. 10.1371/journal.pone.0248671. PMC7972743 33735911

[8] Tahaghoghi-hajghorbani S, Zafari P, Masoumi E, Rajabinejad M, Jafari-shakib R. Since January 2020 Elsevier has created a COVID-19 resource centre with free information in English and Mandarin on the novel coronavirus COVID- 19. The COVID-19 resource centre is hosted on Elsevier Connect , the company ’ s public news and information. 2020.

[9] Kondělková K, Vokurková D, Krejsek J, Borská L, Fiala Z, Andrýs C. Regulatory T cells (Treg) and Their Roles in Immune System with Respect to Immunopathological Disorders. Acta Medica (Hradec Kral Czech Republic). 2010;53(2):73–7. Available from: https://actamedica.lfhk.cuni.cz/53/2/0073/. 10.14712/18059694.2016.63 20672742

[10] Li C, Li J, Ni H. Crosstalk between platelets and microbial pathogens. Front Immunol. 2020. 10.3389/fimmu.2020.01962/full. PMC7426443 32849656

[11] Qin C, Zhou L, Hu Z, Zhang S, Yang S, Tao Y, *et al*. Dysregulation of immune response in patients with coronavirus 2019 (COVID-19) in Wuhan, China. Clin Infect Dis. 2020;71(15):762–8. Available from: https://academic.oup.com/cid/article/71/15/762/5803306 10.1093/cid/ciaa248 PMC7108125 32161940

[12] Bhattacharjee S, Banerjee M. Immune thrombocytopenia secondary to COVID-19: a systematic review. SN Compr Clin Med. 2020;2(11):2048–58. 10.1007/s42399-020-00521-8 PMC7501509 32984764

[13] Qu M, Liu Q, Zhao H-G, Peng J, Ni H, Hou M, *et al*. Low platelet count as risk factor for infections in patients with primary immune thrombocytopenia: a retrospective evaluation. Ann Hematol. 2018;97(9):1701–6. 10.1007/s00277-018-3367-9. PMC6097778 29777278

[14] Papageorgiou C, Jourdi G, Adjambri E, Walborn A, Patel P, Fareed J, *et al*. Disseminated intravascular coagulation: an update on pathogenesis, diagnosis, and therapeutic strategies. Clin Appl Thromb. 2018;24(9_Suppl):8S-28S. 10.1177/1076029618806424 PMC6710154 30296833

[15] Cohn CS. Platelet transfusion refractoriness: how do I diagnose and manage? Hematol (United States). 2020;20(1):527–32. 10.1182/hematology.2020000137 PMC7727584 33275694

[16] Xu P, Zhou Q, Xu J. Mechanism of thrombocytopenia in COVID-19 patients. Ann Hematol. 2020;99(6):1205–8. 10.1007/s00277-020-04019-0 PMC7156897 32296910

[17] Patel T, Stanton N, Gkikas I, Triantafyllopoulou DID. Severe thrombocytopaenia secondary to COVID-19. BMJ Case Rep. 2020;13(9):2–5. 10.1136/bcr-2020-237645 PMC7493096 32933915

[18] Piccinni M-P, Lombardelli L, Logiodice F, Kullolli O, Parronchi P, Romagnani S. How pregnancy can affect autoimmune diseases progression? Clin Mol Allergy. 2016;14(1):11. 10.1186/s12948-016-0048-x. PMC5025626 27651750

[19] Gupta M, Feinberg BB, Burwick RM. Thrombotic microangiopathies of pregnancy: differential diagnosis. pregnancy hypertens. 2018;12:29–34. Available from: https://linkinghub.elsevier.com/retrieve/pii/S2210778917305123. 10.1016/j.preghy.2018.02.007 29674195

[20] Deruelle E, Ben Hadj Salem O, Sep Hieng S, Pichereau C, Outin H, Jamme M. Immune thrombocytopenia in a patient with COVID-19. Int J Hematol. 2020;112(6):883–8. 10.1007/s12185-020-02943-5. PMC7365304 32677007

[21] Hindilerden F, Yonal-Hindilerden I, Sevtap S, Kart-Yasar K. Immune thrombocytopenia in a very elderly patient with COVID-19. Front Med. 2020;7. 10.3389/fmed.2020.00404/full. PMC7365892 32754609

[22] Neunert C, Terrell DR, Arnold DM, Buchanan G, Cines DB, Cooper N, *et al*. American Society of Hematology 2019 guidelines for immune thrombocytopenia. Blood Adv. 2019;3(23):3829–66. 10.1182/bloodadvances.2019000966 PMC6963252 31794604

[23] Mei H, Luo L, Hu Y. Thrombocytopenia and thrombosis in hospitalized patients with COVID-19. J Hematol Oncol. 2020;13(1):13–5. 10.1186/s13045-020-01003-z PMC7705847 33261634

[24] Moores LK, Tritschler T, Brosnahan S, Carrier M, Collen JF, Doerschug K, *et al*. Prevention, diagnosis, and treatment of VTE in patients with coronavirus disease 2019: CHEST guideline and expert panel report. Chest. 2020;158(3):1143–63. 10.1016/j.chest.2020.05.559. PMC7265858 32502594

